# Alcohol, Adipose Tissue and Lipid Dysregulation

**DOI:** 10.3390/biom7010016

**Published:** 2017-02-16

**Authors:** Jennifer L. Steiner, Charles H. Lang

**Affiliations:** Department of Cellular and Molecular Physiology, Penn State College of Medicine, Hershey, PA 17033, USA; jls1075@psu.edu

**Keywords:** lipolysis, lipogenesis, fat, inflammation, white adipose tissue, lipodystrophy

## Abstract

Chronic alcohol consumption perturbs lipid metabolism as it increases adipose tissue lipolysis and leads to ectopic fat deposition within the liver and the development of alcoholic fatty liver disease. In addition to the recognition of the role of adipose tissue derived fatty acids in liver steatosis, alcohol also impacts other functions of adipose tissue and lipid metabolism. Lipid balance in response to long-term alcohol intake favors adipose tissue loss and fatty acid efflux as lipolysis is upregulated and lipogenesis is either slightly decreased or unchanged. Study of the lipolytic and lipogenic pathways has identified several regulatory proteins modulated by alcohol that contribute to these effects. Glucose tolerance of adipose tissue is also impaired by chronic alcohol due to decreased glucose transporter-4 availability at the membrane. As an endocrine organ, white adipose tissue (WAT) releases several adipokines that are negatively modulated following chronic alcohol consumption including adiponectin, leptin, and resistin. When these effects are combined with the enhanced expression of inflammatory mediators that are induced by chronic alcohol, a pro-inflammatory state develops within WAT, contributing to the observed lipodystrophy. Lastly, while chronic alcohol intake may enhance thermogenesis of brown adipose tissue (BAT), definitive mechanistic evidence is currently lacking. Overall, both WAT and BAT depots are impacted by chronic alcohol intake and the resulting lipodystrophy contributes to fat accumulation in peripheral organs, thereby enhancing the pathological state accompanying chronic alcohol use disorder.

## 1. Introduction

There is growing appreciation of the role of adipose tissue as a key regulator of whole-body metabolic control with actions that extend far beyond that of just an inert energy storage organ (as reviewed in [1]). Its role as an endocrine organ is well-recognized and the importance of the proteins it synthesizes and secretes (i.e., adipokines) continues to expand. In addition to the release and actions of adipokines like leptin and adiponectin, adipose tissue is essential to glucose homeostasis as a primary site for glucose uptake. As the largest energy storage depot in the body, adipose tissue is also intimately linked with energy utilization and provision in response to changes in nutritional state. For example, fasting as well as inflammation, activate lipolysis leading to the release of stored fatty acids from adipose tissue [2]. Conversely, energy storage is enhanced during times of excess intake. Therefore, adipose tissue mass is dictated by the long-term balance between lipogenesis and lipolysis. Perturbation of this balance during diseases such as obesity when pathological expansion occurs can lead to ectopic lipid storage in peripheral tissues including the liver and skeletal muscle. Such disease-related changes in adipose tissue include increased inflammation and aberrant adipokine secretion in conjunction with hypoxia, apoptosis, and oxidative stress [3,4].

It has long been recognized that alcohol (i.e., ethanol) consumption perturbs lipid metabolism causing adipose tissue dysfunction [5,6]; however, interest has been renewed after it was definitively shown that fatty acids released by adipose tissue are transported to the liver and contribute to hepatic steatosis [7,8]. Therefore, while we have not focused on discussing changes in liver health, it is noteworthy that changes in the inflammatory environment and lipid balance have the potential to impact the susceptibility to liver disease because of the extensive crosstalk between these two organs. Further, to maintain our focus primarily on adipose tissue, we have not included changes in circulating levels of cholesterol, triglycerides or inflammatory mediators, as many tissues can contribute to the flux of these molecules in response to alcohol.

The purpose of this review is to highlight the current knowledge related to the regulation of adipose tissue function by alcohol. After discussing the effects of alcohol on adipose tissue mass, the regulation of lipid balance in regards to lipolysis and lipogenesis will be presented. As an inhibitor of lipolysis, activator of lipogenesis, and important mediator of glycemic balance, the role of insulin will be highlighted in these sections. Adipokines including adiponectin, leptin, resistin and several inflammatory mediators (tumor necrosis factor alpha (TNFα), interleukin-6 (IL-6), monocyte chemoattractant protein-1 (MCP-1)) may also be important modulators of lipid metabolism following chronic alcohol intake and will therefore be discussed. All data presented within these initial sections, unless otherwise specified, will be specific to white adipose tissue (WAT); however, in the last section the effects of alcohol on brown adipose tissue (BAT) and thermogenesis are described. Methodological differences will be noted when appropriate, including variations in feeding paradigms and sex of animals/participants. Of note, the majority of the work reported has been performed in male animals that have consumed alcohol chronically (more than 1 week) as many of the detrimental effects, including lipid deposition in the liver, require prolonged intake to recapitulate the effects observed in humans. While the effects of acute intoxication are discussed when evidence exists, its role in long-term adipocyte dysfunction remains undefined.

## 2. Chronic Alcohol and Adipose Tissue Mass

Changes in adipose tissue depot size are reflective of the net metabolic effects of chronic alcohol consumption including the balance between lipolysis and lipogenesis. In mice, the preponderance of data show that chronic alcohol decreases the weight of epididymal white adipose tissue (eWAT) [7,9,10,11,12,13,14,15], subcutaneous white adipose tissue (sWAT) [10,12], perirenal adipose tissue [7], mesenteric adipose tissue [7], and, correspondingly, adipocyte size [7,11,13,14]. The only report of an increase in perigonadal fat was in female mice, compared to all of the former studies that were performed in males, suggesting a possible sexual dimorphic response that requires further investigation. In contrast, chronic alcohol feeding in rats does not consistently increase fat mass potentially due to improper pair-feeding techniques [16,17], differences in macronutrient composition of the diets (i.e., high fat versus low fat), doses of alcohol used, feeding methods (liquid diet, in water, through gastric tube), or the interaction of these methodological variances in the different adipose tissue depots (eWAT vs. sWAT). Of those animals receiving alcohol as part of a complete nutritionally adequate liquid diet, visceral and subcutaneous adipose tissue mass was unchanged [18,19], while eWAT was either unchanged [20] or decreased [19,21]. A high dose of alcohol (5 g/kg/day) administered via a gastric tube to animals consuming a low fat (10%) chow diet increased eWAT and perirenal depots [22]. On the other hand, lower doses (2.5 and 0.5 g/kg/day) using this same feeding model did not change adipose tissue weight [22,23,24]. Additionally, feeding 5 g/kg/day of alcohol along with a high fat diet (59% fat) prevented the high fat diet induced increase in eWAT mass, suggesting that dietary fat composition has the potential to modulate the alcoholic effect [25].

Similar to the effects of long-term alcohol intake in mice, chronic alcoholics or those regularly consuming alcohol tend to have decreased fat mass [26,27,28,29], while body weight or body mass index (BMI) is unchanged [26,30], and waist to hip ratio is increased [29,31,32,33,34,35], indicating a redistribution of fat to central regions. Interventional studies in which participants consumed moderate amounts of alcoholic beverages each night for several weeks either did not change body weight [36] or slightly increased it [37], suggesting loss in body fat is likely dose- and time-dependent, requiring more prolonged, higher level alcohol intake. Lastly, in a retrospective analysis, beer consumption was not related to obesity and wine consumption was negatively associated with fatness and percent body fat in women, and BMI in men [38]. Therefore, in a healthy population, low to moderate alcohol intake is more likely to increase fat mass while heavy chronic intake leads to pathological lipoatrophy or redistribution via alteration in lipolytic and lipogenic balance as highlighted in the following section.

## 3. Regulation of Lipid Balance

### 3.1. Lipolysis

The balance between lipolysis and lipogenesis dictates adipose tissue mass in response to alterations in nutrient availability and/or the development of pathological conditions. Lipolysis entails triglyceride hydrolysis to fatty acids and glycerol for use as energy by other tissues during times of fasting or inflammation. Chronic alcohol intake activates adipose tissue lipolysis and increases the release of free fatty acids (FFA) [7,8,9,11,39,40]; however, circulating levels of FFA may not always reflect this change as they are removed by other tissues (e.g., liver, heart). While in vivo metabolic flux studies using radiolabeled triglycerides have definitively shown that chronic alcohol consumption increases lipolysis [8,40], primary adipocytes from chronic alcohol-fed rats revealed that basal rates of lipolysis and FFA release were not altered by alcohol feeding [39,40,41]. Sympathetic nervous system activation resulting in catecholamine (epinephrine, norepinephrine) stimulation of β-adrenergic receptor signaling is a potent activator of lipolysis, while insulin has inhibitory actions. Early work provided evidence of the effect of alcohol on epinephrine-stimulated lipolysis as well as insulin-mediated suppression [42,43]. Itaya et al., 1978 reported that alcohol did not alter the release of FFA after stimulation with epinephrine in rat WAT [43], while Yki-Järvinen et al., 1987 showed that alcohol infusion in healthy men prevented the insulin-mediated suppression of plasma triglycerides, suggesting alcohol antagonized the inhibitory actions of insulin on lipolysis [42]. Additionally, Frayn et al., 1990 presented data showing that acute alcohol increased lipolysis based on the measurement of FFA and glycerol, as well as carbon exchange across adipose tissue using arterial-venous differences [44]. These studies laid the groundwork for later mechanistic investigations that revealed the ability of alcohol to increase lipolysis resulted principally from the inhibitory actions of insulin rather than the stimulatory actions of catecholamines on lipolysis [41]. Contradictorily, catecholamine-mediated lipolysis was decreased by alcohol in cultured adipocytes from alcohol-fed rats [39,40]. As alcohol increases the net lipolytic rate, its ability to dis-inhibit the effect of insulin must supersede the alcohol-mediated downregulation of β-adrenergic receptor (β-AR) signaling to result in an increase, not decrease, in lipolysis.

**Figure 1:**
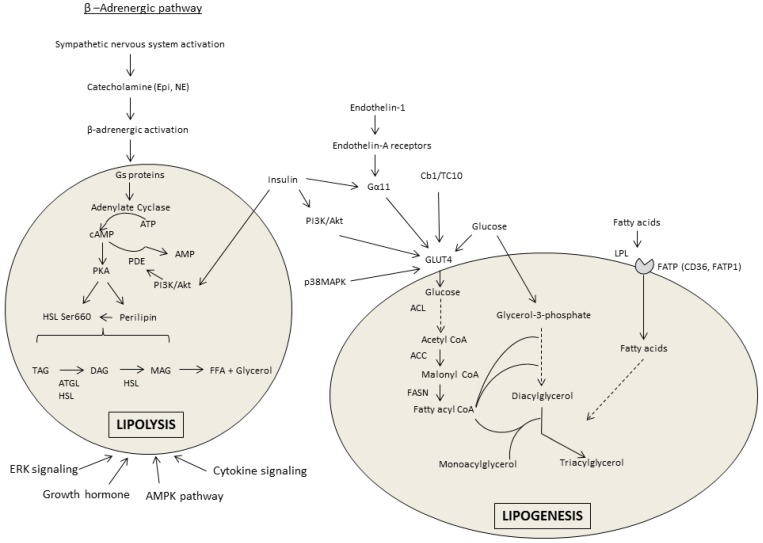
Overview of the lipolytic and lipogenic pathways in white adipose tissue. Lipolysis is predominantly activated by the β-adrenergic signaling pathway that increased the release of free fatty acids (FFA) and glycerol. Lipogenesis is stimulated by insulin and enhances the uptake of FFA and synthesis and storage of triacylglycerol (TAG). The shaded areas represent adipocytes. Abbreviations: epinephrine (Epi); norepinephrine (NE); cyclic adenosine monophosphate (cAMP); adenosine triphosphate (ATP); phosphodiesterase (PDE); protein kinase A (PKA); hormone sensitive lipase (HSL); phosphoinositide 3-kinase (PI3K); adipose tissue triglyceride lipase (ATGL); diacylglycerol (DAG); monoacylglycerol (MAG); extracellular signal related kinase (ERK); adenosine monophosphate kinase (AMPK); ATP-citrate lyase (ACL); Acetyl Coenzyme A (CoA) carboxylase (ACC); fatty acid synthase (FASN); fatty acid transport proteins (FATPs); lipoprotein lipase (LPL); mitogen activated protein kinase (MAPK); cluster of differentiation 36 (CD36); glucose transporter-4 (GLUT4).

Within the β-AR stimulatory pathway of lipolysis there are several regulatory points altered by alcohol; however, differences exist between the investigations reporting a decrease in β-AR stimulated lipolysis and those reporting an increase in response to chronic alcohol treatment (see Figure 1 for pathway overview). These differences could potentially be model system-dependent, as culturing adipocytes from the alcohol-treated rats might alter signaling compared with that measured directly in the tissue which showed lipolysis to be increased.

Upon stimulation of the β-adrenergic pathway, G proteins mediate the increased conversion of adenosine triphosphate (ATP) to cyclic adenosine monophosphate (cAMP) via adenylate cyclase. Basal levels of cAMP in WAT are increased [14,45,46,47] or unchanged [20,39,45,47] by chronic alcohol consumption. Interestingly, the fat content of the diet consumed influences cAMP accumulation independent of the alcohol content. A high fat liquid diet (35% kcal from fat) alone enhanced cAMP accumulation and lipolytic enzyme expression compared with a standard rodent chow diet [45]. In work where β-AR lipolysis was suppressed by alcohol, the isoproterenol-mediated induction of cAMP is also decreased in relation to stimulation of phosphodiesterase 4 (PDE4) activity that increases cAMP breakdown to AMP [39]. Accordingly, basal PDE4 activity was increased after chronic alcohol and inhibition of PDE4 using R020-1724 prevented the effect of alcohol on cAMP [39]. Stimulation of protein kinase A (PKA) via cAMP after isoproterenol treatment of isolated primary adipocytes from chronic alcohol-fed rats was impaired despite basal activity remaining unchanged [39]. PKA phosphorylates proteins required for the induction of lipolysis, including perilipin and hormone sensitive lipase (HSL). Perilipin A resides on the surface of the lipid droplet and acts as a barrier to lipolysis induction until it is activated by PKA. Upon activation, the conformational change in perilipin allows for movement of HSL to the surface where it can contact triglycerides and enhance hydrolysis. Perilipin protein (but not mRNA) was increased in eWAT isolated from mice undergoing a 12-day chronic-binge protocol [14]; however, perilipin phosphorylation was decreased after catecholamine stimulation in primary adipocytes from alcohol-fed rats [39].

PKA phosphorylates HSL on Ser660 thereby increasing its activity and the removal of fatty acids from diacylglycerol (DAG) to form monoacylglycerol (MAG). HSL phosphorylation after chronic alcohol appears to vary between models and conditions. Consistent with their findings that alcohol suppresses lipolysis via the β-AR pathway [39,40], Kang et al., 2006 found that alcohol decreases HSL Ser660 phosphorylation [39]. Only one other investigation has reported a similar decrease in eWAT from rats after 12 weeks of an alcohol-containing liquid diet [19]. Instead, the majority of work shows HSL phosphorylation, activity or mRNA to be increased [7,9,11,12,14,15,18,45,46] or at least unchanged by alcohol [7,14,18,45]. The preponderance of data indicate that chronic alcohol feeding increases adipose tissue triglyceride lipase (ATGL), the other rate-limiting enzyme in adipose tissue lipolysis, that catalyzes the removal of the first fatty acid from triacylglycerol [7,9,12,14,15,18,19].

Insulin inhibits lipolysis to promote fat storage. In this regard, insulin can enhance phosphodiesterase-3B (PDE3B) activity to decrease PKA and suppress lipolysis. However, PDE3B activity, mRNA, and protein are unchanged in isolated adipocytes from chronic alcohol fed rats [40], and decreased in mice after 5 weeks of alcohol intake (20% *w*/*v* in drinking water) [46], suggesting this is an unlikely mechanism. Insulin can also phosphorylate protein phosphatase 1 (PP1), which then dephosphorylates HSL to impair lipolysis. Chronic alcohol intake inhibited PP1 phosphorylation, suggesting a possible mechanism by which alcohol can overcome the insulin-mediated suppression of lipolysis [46].

Adipose tissue lipolysis can also be regulated by fibroblast growth factor 21 (FGF21) that stimulates lipolysis independent of catecholamine activation and suppresses lipid accumulation via peroxisome proliferator-activated receptor gamma (PPARγ) and CCAT-enhancer-binding protein (C/EBP) (discussed further below). FGF21 is an energy-responsive adipokine that is only secreted from WAT after feeding, and accordingly, glucose uptake, fatty acid synthesis and PPARγ activation in adipose tissue [48]. Chronic-binge alcohol increases both plasma and eWAT FGF21 expression and in mice deficient for FGF21 the alcohol-mediated increase in lipolysis (measured by the appearance of FFA and glycerol) was prevented [14]. Further, the alcohol-induced decrease in eWAT mass was reduced in FGF21 knockout (KO) mice compared to wildtype animals. Based on an increase in plasma catecholamine concentration, but not insulin, in wildtype and not KO mice, Zhao et al., [14] posited that FGF21 promoted alcohol-induced lipolysis through activation of the β-adrenergic pathway. As this is in direct contradiction to the evidence presented above [39,40], it is clear that the role of FGF21 during alcohol intoxication requires further investigation as its importance as a metabolic regulator continues to expand.

### 3.2. Lipogenesis

In opposition to lipolysis, lipogenesis occurs in the adipocyte during times of energy surplus. While the lipolytic effects of alcohol outweigh its lipogenic effects, alcohol does modulate several components of the lipogenic pathway (see Figure 1 for pathway overview). For example, PPARγ which is among the most prominent lipogenic stimulators is generally decreased in WAT after chronic alcohol consumption [7,9,12,15,18,19,49,50]. Treatment with the PPARγ agonist rosiglitazone restored this alcohol-mediated perturbation [12,51]. The mitogen-activated protein kinase (MAPK) pathway regulates PPARγ and inhibition of MAPK partially restored PPARγ levels decreased by alcohol [49]. Through its activation and joint binding with PPARγ, C/EBPα is central to the stimulation of adipogenesis and adipocyte differentiation. Chronic alcohol intake decreased C/EBPα expression in eWAT [7,15,50], although no change was observed in sWAT or in 3T3-L1 cultured with alcohol [19]. Lipin1, another PPARγ regulator, is similarly decreased in eWAT of chronic alcohol-fed rats [19], but unchanged in sWAT and in cultured adipocytes [19].

The principle enzyme regulating lipogenesis is lipoprotein lipase (LPL) that enables lipoprotein uptake as well as hydrolysis within the adipocyte. Expression of LPL is decreased by alcohol in a chronic-binge model or not changed [9,51] in vitro and in vivo [47,52]. Members of the fatty acid transport protein family, cluster of differentiation 36 (CD36) and fatty acid transport protein-1 (FATP1), that transport fatty acids inside the cell, are unchanged in a chronic-binge model [9] or after 8 weeks of alcohol feeding [7]. Therefore, alcohol appears to generally decrease lipogenic signaling in animal models of chronic alcohol intake.

Insulin is not only important for lipolysis suppression but also for stimulating lipogenesis by increasing both fatty acid and glucose uptake. Glucose is a direct substrate for fatty acids via downstream conversion to either glycerol-3-phosphate or acetyl Coenzyme A (CoA). The conversion of glucose to fatty acyl CoA (FA-CoA) requires several regulatory enzymes, many of which are modulated by chronic alcohol. First, the phosphorylation of ATP-citrate lyase (ACL), which generates acetyl CoA from citrate (product of glucose metabolism), is decreased by alcohol in eWAT (but not sWAT) of rats and primary adipocytes [19]. Acetyl CoA carboxylase (ACC) catalyzes the conversion of acetyl CoA to malonyl CoA. Alcohol suppresses ACC Ser79 phosphorylation in vivo [9,18,19,23,25], while in vitro no change and an increase is observed depending on the dose of alcohol [19,25]. This decrease in ACC phosphorylation is consistent with the concomitant reduction in activity of its protein kinase, AMP kinase (AMPK), observed after chronic alcohol consumption [9,18,23,25]. As AMPK activation in adipose tissue can also suppress lipolysis (basal and stimulated), the alcohol-related decrease in AMPK may similarly lead to enhanced lipolysis.

Fatty acid synthase (FASN) catalyzes the conversion of malonyl CoA to fatty acyl CoA in preparation for incorporation into the growing triacylglyceride molecule. The alcohol-induced decreased in FASN in eWAT [12,19] is prevented by rosiglitazone indicating the importance of PPARγ in the regulation of several lipogenic proteins. However, there are contrasting reports of FASN expression, including no change in cultured adipocytes or sWAT [19], suggesting that alcohol may have adipose tissue depot-specific effects on lipid metabolism. After the addition of the FA-CoA molecules to either monoacylglycerol (MAG) or glycerol-3-phosphate, diacylglycerol acyl transferase (DGAT) catalyzes the addition of the last FA-CoA to form the triglyceride. DGAT mRNA content in eWAT of mice was unchanged [7] or decreased [12] by chronic alcohol. Lastly, in isolated adipocytes, alcohol and acetaldehyde only decreased insulin-induced lipogenesis at supra-physiological concentrations [53]. Overall, the majority of findings support the potential for an alcohol-induced decrease in several regulatory proteins, although the effects of chronic alcohol intake on lipogenic modulators may vary based on the model system (in vivo vs. in vitro). Whether those decreases summate to result in an overall decrease in lipogenesis remains unknown, although in vivo administration of labeled triglycerides to chronically alcohol-fed rats did indicate no significant change in the rate of triglyceride synthesis.

### 3.3. Acute Alcohol and Lipid Balance

The effects of chronic alcohol on lipid balance have been investigated more extensively than the effects of a single dose of alcohol. Somewhat contradictory to the long-term animal studies reported above, Siler et al., 1999 [54] showed that acute alcohol intake of 24 g in healthy men decreased whole body lipid oxidation, the concentration of FFA in the plasma and the appearance of glycerol, indicating either no effect or a decrease of lipolysis and a potential increase in lipogenesis [55,56]. In support of a lack of an acute effect, the activity of HSL and LPL, and cAMP accumulation were unaltered after acute intoxication in mice [46,47]. The data on the acute effects of alcohol on adipose tissue lipolysis are therefore extremely limited but merit further study as early changes produced by alcohol may provide insight into the initiating events of long-term alcohol-induced lipodystrophy.

### 3.4. Adipose Tissue-Liver Crosstalk

Impaired lipid storage in adipose tissue may lead to fat deposition in liver and other organs throughout the body. Accordingly, in alcoholics, a decrease in fat mass has been associated with enhanced liver fat [28]. Recently, the use of stable isotopes has revealed that fatty acids released from adipose tissue in response to chronic alcohol consumption are transported to the liver where they accumulate via what has been termed the “reverse triglyceride transport” mechanism [7,8]. Alcohol also modulates the expression of lipoprotein receptors in the liver to regulate uptake of fatty acids released from the adipose tissue. Accordingly, chronic alcohol intake increased CD36 [7,57,58,59] and very low density lipoprotein receptor (VLDLR) expression in the liver [60], whereas knock-out of these receptors protected mice from the development of alcohol-induced fatty liver [57,60]. In contrast, chronic alcohol intake decreased VLDLR and LPL [60] and did not change CD36 expression in WAT [7], indicating differential regulation between tissues and potentiation of the lipolytic phenotype induced by alcohol. In contrast, the effects of alcohol on the expression of FATPs in the liver are inconsistent and differ with each form. FATP1 has been reported to be increased [61], decreased [58], and unchanged [57,59]; similarly, changes in FATP2 and FATP5 are also variable within and across studies in chronic alcohol-fed animals [7,57,58,59,61].

Further, triglycerides taken up by the liver are not solely derived from adipose tissue as fatty acids from both the diet and those synthesized de novo can contribute to hepatic lipid accumulation [8]. Wei et al., 2013 also showed that the amount of labeled triglycerides in eWAT and sWAT decreased over a four-week alcohol feeding period in mice, indicating increased lipolysis in both fat depots [8]. The type of lipid included in the diet may be important in the stimulation of lipolysis by alcohol resulting in steatosis. That is, providing a diet comprised of flax oil that is rich in omega-3 polyunsaturated fatty acids ameliorated the effects of alcohol on adipose tissue lipolysis and lessened the development of hepatic steatosis [9]. As discussed further below, adipokines including leptin and adiponectin are implicated in the development of fatty liver disease and treatment with either leptin [10] or adiponectin [62] reversed alcohol-induced fatty liver.

### 3.5. Glucose Homeostasis in Adipose Tissue

It is well-recognized and reviewed elsewhere [63] that chronic alcohol intake can impair insulin action and glucose tolerance; therefore, changes in these measures will not be reiterated herein [7,22,23,33,64]. However, adipose tissue, in addition to liver and skeletal muscle, is a primary site of glucose disposal. The preponderance of data indicate that chronic alcohol intake perturbs insulin-stimulated (but not basal) glucose uptake in adipose tissue [15,17,20,41,64,65]. Remarkably, alcohol does not perturb insulin stimulation of phosphoinositide-3-kinase (PI3K)/Akt signaling in the dysregulation of glucose uptake, nor does it alter the mRNA of insulin receptor, insulin receptor substrate-1, or PP1 [65]. The full effect of alcohol on insulin-mediated glucose uptake also cannot be ascribed to the alcohol-mediated increase in G_s_α content at the membrane and cAMP accumulation that decrease glucose transporter-4 (GLUT4) [20,25], as the magnitude of this effect did not match the percent inhibition [20]. Further, as neither translocation of intracellular GLUT4 vesicles to the plasma membrane or expression of GLUT4 trafficking and docking proteins were altered by chronic alcohol, it was ultimately determined that alcohol prevented the insulin-mediated increase in GLUT4 vesicular fusion and therefore accessibility at the surface of the adipocyte [65]. Other studies also showed that chronic alcohol decreases myocyte enhancer factor 2 (MEF2), a binding site of the GLUT4 promoter, in an AMPK-dependent manner [23,25]. Acutely, alcohol (2.5 g/kg/day, 3 days) decreased insulin-stimulated phosphorylation of Akt Ser473 in mesentery but not subcutaneous adipose tissue, although this was the only measure of insulin sensitivity reported [66].

Although the insulin-stimulated increase in GLUT4-mediated glucose uptake most commonly occurs via PI3K signaling, other pathways contribute. For instance, endothelin-1 also utilizes G-protein signaling in the regulation of glucose uptake. Chronic alcohol impairs this pathway likely by decreasing Gα_11_ and preventing endothelin-1 stimulated tyrosine phosphorylation of proline rich tyrosine kinase 2 [67]. Alternatively, chronic alcohol can also disrupt the Cb1/TC10 pathway at multiple sites in relation to its impairment of glucose uptake within adipose tissue [68].

### 3.6. Mammalian Target of Rapamycin Activity in Adipose Tissue

**Figure 2:**
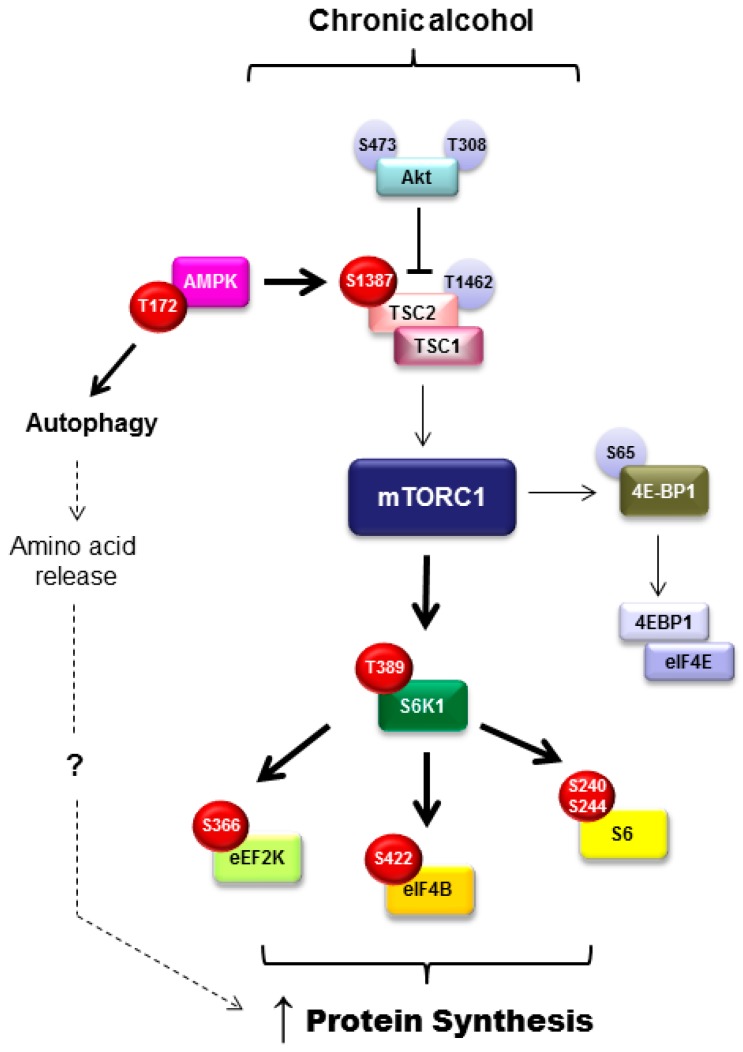
Effect of chronic alcohol intake on mammalian/mechanistic target of rapamycin complex 1 (mTORC1) signaling in white adipose tissue from mice. Bold arrows indicate activation of selected pathway components; red circles indicate an alcohol-induced increased in phosphorylation and the presumed activation of respective substrates; light blue circle indicates phosphorylation site not altered by alcohol; hashed lines indicated a hypothesized mechanism. Abbreviations: threonine (T) of given phosphorylation site; serine (S) of indicated phosphorylation site; tuberous sclerosis complex 1/2 (TSC); eukaryotic initiation factor 4E (eIF4E) binding protein 1 (4E-BP1); ribosomal protein S6 kinase (S6K1); ribosomal protein S6 (S6); elongation factor 2 kinase (eEF2K); and eukaryotic initiation factor 4B (eIF4B).

An additional regulator of adipose tissue metabolism including adipogenesis, lipolysis, and lipogenesis is the mammalian/mechanistic target of rapamycin complex 1 (mTORC1). Adipogenesis is positively regulated by mTORC1 and differentiation is impaired upon mTOR inhibition [69]. mTORC1 signals to several different proteins in the control in adipose tissue function. For example, inhibition of eukaryotic translation initiation factor 4E-binding proteins (4E-BP) by mTORC1 promotes adipocyte maturation and suppression of lipolysis via early response transcription factor, which then blocks ATGL expression. Further, LPL and triglyceride hydrolysis are inhibited by mTORC1 signaling, indicating suppression of lipolysis [69]. With this in mind and the knowledge that chronic alcohol intake suppresses mTORC1-mediated protein synthesis within skeletal muscle [70], it was unexpected that mTORC1 signaling and rates of protein synthesis were increased concomitant with the loss in fat mass after chronic alcohol feeding in mice (refer to Figure 2 for a more detailed overview of those signaling proteins modulated by alcohol) [15]. As protein is a small component of the adipocyte, it is unlikely that changes in protein synthesis would completely explain the alcohol-mediated loss in adipose tissue weight; however, this work showed no relationship between those variables. Alternatively, alcohol may induce a state of hyperactive and aberrant mTOR signaling similar to that seen during obesity. As in obesity, this may contribute to an increase in inflammatory mediators (TNFα and IL-6), a decrease in adiponectin, and potentially insulin resistance [15].

In addition to the increase in protein synthesis, alcohol also increased autophagy in an mTORC1-independent and AMPK-dependent manner [15]. It was posited that the alcohol-induced increase in autophagy contributes to the contradictory enhancement of protein synthesis by increasing intracellular levels of amino acids. Acute alcohol did not evoke the same induction of autophagy, despite increasing protein synthesis, suggesting that other mechanistic explanations exist related to the alcohol-induced increase in adipose tissue protein synthesis. Further, as these events were not recapitulated in cultured 3T3-L1 adipocytes, they do not appear to represent a direct effect of alcohol on the adipocyte [15]. As this is the first report of alcohol-induced changes in protein balance and mTORC1 signaling within adipose tissue, additional work in this area is required to determine how these changes contribute to overall lipid balance in alcohol use disorders.

## 4. Alcohol Metabolism

The byproducts of alcohol metabolism are implicated in its deleterious effects throughout the body. Alcohol is first metabolized to acetaldehyde by either alcohol dehydrogenase (ADH) or the cytochrome P450 2E1 (CYP2E1) system. CYP2E1 is expressed in eWAT, sWAT, and 3T3-L1 adipocytes (with relatively greater amounts found in sWAT) and metabolism through this system can lead to oxidative stress and DNA damage [19,21,31]. Alcohol intake either upregulates CYP2E1 [71] or does not change its expression in adipose tissue [19,31]. In a proof of concept experiment, overexpression of CYP2E1 in 3T3-L1 adipocytes incubated with alcohol decreased adiponectin secretion similar to that seen in vivo in rodents [21]. ADH is primarily expressed in the liver, but also found at low levels in eWAT and BAT and is almost undetectable in sWAT [19,72]. Although ADH expression was unchanged in chronic alcohol-fed mice, it may still be important in the production of acetaldehyde and the effects of that metabolite on WAT [19].

Chronic alcohol increases circulating concentrations of acetaldehyde that cause many of the detrimental effects of alcohol. Culturing 3T3-L1 adipocytes with acetaldehyde, but not alcohol, decreased lipogenic regulators including PPARγ, C/EBP, ACC, FASN, Lipin, and diacylglycerol *O*-acyltransferase-2 (DGAT2) [19]. Further, blocking acetaldehyde metabolism markedly decreased these lipogenic enzymes presumably due to the increased concentration of acetaldehyde [19]. Aldehyde dehydrogenase (ALDH), which catalyzes the production of acetate from acetaldehyde, is expressed in eWAT and sWAT and activity is increased in chronic alcohol-fed mice [19]. Protein expression of ALDH3A1 and ALDH1A1, contributing to the clearance of aldehyde, is increased in eWAT in response to chronic alcohol but unchanged in sWAT despite higher basal levels [19]. The alcohol-induced increase in these enzymes may suggest an attempt to clear the toxic metabolite. Further, it has been hypothesized that depot-specific expression of these enzymes may be related to the differing lipogenic effects of alcohol [19].

The lack of expression of these enzymes within adipocytes may also contribute to the differences observed between in vivo and in vitro models following alcohol. Adipocytes treated with alcohol ex vivo, instead of being isolated from alcohol-fed animals, do not appear to recapitulate the alcoholic condition with high fidelity. For example, exposure of 3T3-L1 adipocytes to alcohol did not alter lipolysis (median glycerol levels), lipogenic mediators (discussed above), or protein synthetic signaling [14,15,19]. Therefore, either treatment with acetaldehyde which bypasses the need for alcohol metabolism, or primary adipocytes from alcohol-treated animals should be utilized to investigate the adipose tissue and lipid metabolism specific effects of alcohol.

## 5. Adipokines

White adipose tissue is an endocrine organ secreting more than 600 different proteins or adipokines, which regulate metabolism within multiple tissues including, liver, skeletal muscle, and brain [73]. Leptin and adiponectin are among the primary adipokines measured and accordingly, several investigations have implicated these adipokines in the development of alcoholic lipodystrophy and hepatic steatosis.

### 5.1. Adiponectin

Adiponectin is an anti-inflammatory adipokine with adipogenic and insulin sensitizing effects via modulation of AMPK signaling, affecting fatty acid oxidation and glucose uptake in peripheral tissues. The promotion of appropriate lipid storage and adipogenesis by adiponectin is central in the prevention of ectopic fat storage. It is not surprising then that the majority of data garnered from animal models of chronic alcohol consumption show circulating adiponectin to be decreased [9,18,49,50,51,62,74,75,76,77], although a few report no change [10,12,25,78]. As little as four days of an alcohol-containing liquid diet was sufficient to decrease adiponectin levels [50]. This inhibitory effect was independent of the animal species (mice, rats, micropigs, macaques) as well as the mode of alcohol ingestion (in drinking water or liquid diet).

Conversely, adiponectin measured in the serum of humans was increased in relation to alcohol consumption [36,37,79,80,81,82,83,84], although two investigations did report a dose-dependent decrease [34,35]. When alcohol (beer, wine, or whisky) was added to the diet at a low to moderate level for a short period of time (2.5, 3 or 6 weeks) serum adiponectin levels were increased at the end of the intervention period [36,37,80,84]. Similarly, retrospective studies indicated that alcohol intake was associated with higher serum adiponectin levels in men and women [33,82]. Despite a decrease in adiponectin in chronic heavy drinkers (>50 g/day), no correlative relationship between adiponectin and daily alcohol intake was found [35] and controlling for metabolic syndrome eliminated this relationship [34]. These results suggest that higher doses of alcohol given to animals likely contributed to the decrease in adiponectin, while in humans, lower doses of alcohol appear to improve adiponectin status, although a definitive explanation for this apparent species-specific response has not been elucidated. Pertaining to this discrepancy, despite being an abundant producer of adiponectin, the contribution of bone marrow adipose tissue to adiponectin secretion is rarely measured.

Additionally, there are three circulating oligomeric complexes of adiponectin, including high-, middle- and low-molecular weight adiponectin, although few studies measure levels of these different complexes in response to alcohol. In a murine model, higher levels of alcohol intake decreased both the high- and middle-molecular forms [50], with rosiglitazone treatment normalizing the alcohol-induced decrease [51]. Conversely, moderate alcohol intake in women and men increased the high molecular weight form [37,80,85] and to a lesser degree the middle-molecular weight, while the low molecular weight form was not changed [80]. Therefore, it appears that alcohol-induced changes in the different forms are similar to those changes observed in total adiponectin.

Paralleling the decrease in circulating adiponectin, all but one study [16] showed that adiponectin mRNA or protein in adipose tissue was suppressed following chronic alcohol feeding [15,18,24,25,49,51,74,86]. While these findings include several models and fat depots, one investigation did report depot-specific effects in that adiponectin secretion from retroperitoneal and subcutaneous adipocytes were suppressed while secretion from eWAT was unaffected by alcohol [86]. In contrast, adiponectin mRNA was increased in human subcutaneous fat biopsies from women after 6 weeks of moderate wine intake [37]. In support of this discrepant finding, culturing human subcutaneous adipose tissue with alcohol also dose-dependently (11, 22, 44, 88 mM alcohol) increased adiponectin mRNA, protein, and synthesis [87]. Further, cessation of alcohol intake for 1 week in chronic alcoholic patients decreased mRNA expression of adiponectin and its receptors within adipose tissue [88].

Although a lack of congruency between animal and human models for alcohol-induced changes in adiponectin exist, potential mechanisms have been identified for the decrease seen in rodents. Using cultured visceral adipose tissue cells from rat, it was determined that upregulation of the MAPK pathway and suppression of PPARγ by alcohol decreases adiponectin secretion [49]. Cellular stress resulting from chronic alcohol intake also contributes to the impairment in adiponectin release in alcohol-treated animals. First, upregulation of CYP2E1 after alcohol feeding was associated with a decrease in adiponectin and subsequent induction of oxidative stress, including enhanced accumulation of 4-hydroxynonenol (4-HNE) and a decreased glutathione (GSH/GSSG) ratio [21,50]. Increased 4-HNE and suppressed catalase activity was seen after as little as 4 days of an alcohol-containing diet [50], consistent with a decrease in adiponectin, and highlighting the immediate impact of alcohol on this adipokine. The oxidative stress-related decrease in adiponectin can be remediated by supplementation with the amino acid taurine, as levels are depleted by chronic alcohol intake [50].

While no evidence of an alcohol-mediated induction of endoplasmic reticulum (ER) stress markers (splicing of X-box binding protein 1 (XBP) mRNA, eukaryotic initiation factor 2 alpha (eIF2α) or CCAAT-enhancer-binding protein homologous protein (CHOP)) was reported at early time points (4 and 7 days) [50], a separate investigation showed that four weeks of alcohol intake increased CHOP mRNA in eWAT in association with a decrease in adiponectin [24]. Here the suppression of adiponectin and associated increase in ER stress was related to an alcohol-induced increase in homocysteine levels in eWAT [11,74]. The increase in homocysteine was accompanied by a decrease in methylation potential (i.e., *S*-adenosylmethionine (SAM)/*S*-adenosylhomocysteine (SAH) ratio) and the enzyme cystathionine β-synthase that catalyzes the conversion of homocysteine to cysteine. Remediation of homocysteine metabolism by treatment with betaine normalized plasma adiponectin and lipolysis altered by chronic alcohol consumption [11,74].

All of the work presented thus far pertains to the chronic effect of alcohol on adiponectin, as little work has reported the acute effects of alcohol on this adipokine. A single dose of alcohol decreased circulating levels at 30 min (but not 24 h) post-administration in rats [89], whereas adiponectin mRNA was unchanged in a chronic-binge murine model (3 days alcohol, 2–4 day rest, 3 days alcohol) [90]. Lastly, in a repeated binge-like alcohol paradigm (2.5 g/kg/day, 3 days) serum adiponectin was decreased in the rats [66]. Due to the limited data, definitive conclusions cannot be made with any confidence regarding the acute effects of alcohol on adiponectin.

### 5.2. Leptin

Leptin regulates several processes including food intake, energy expenditure, lipolysis, fatty acid oxidation, lipogenesis, and insulin sensitivity. Leptin receptors are found on several tissues throughout the body in addition to adipose tissue, indicating both paracrine and autocrine functions for the hormone. For example, leptin inhibits lipogenesis and activates β-oxidation of fatty acids in liver and thereby can potentially reduce lipid deposition [73]. Unlike adiponectin, the effects of alcohol on circulating leptin are less consistent and may be related to changes in fat mass instead of the alcohol per se. Leptin has been reported to be increased [24,91,92], decreased [10,93,94], and unchanged [24] across a range of rodent models of chronic alcohol intake. Further, administration of leptin in combination with alcohol decreased body weight and eWAT mass suggesting that increased leptin may decrease appetite or increase energy expenditure [10]. Consideration of potential methodological differences between these divergent findings presents no clear pattern or consistencies. However, while leptin is sensitive to several physiological stimuli including feeding status (fasted vs. fed) and body weight, nutrient status was infrequently reported or controlled for and correlations between body weight and leptin were not always performed. Similar to the inconclusive results reported for circulating leptin in animal models, the serum leptin concentration from humans appears to be unrelated to alcohol intake [80,87,95,96,97,98,99], although exceptions do exist [27,100,101,102]. In alcoholic patients, leptin has been reported to be increased, decreased or unchanged, and serum leptin was also not altered by either alcohol withdrawal for 15 days [27] or the severity of liver disease [87,95,96].

An increase in leptin protein [16], mRNA [22,24,103], and its receptor [91] is detected within adipose tissue of chronic alcohol-fed rats and mice. In contrast, leptin mRNA was unchanged in subcutaneous adipose tissue from alcoholic patients; however, differences in adipose tissue depot may have contributed to these discrepant findings [30]. Acutely, alcohol increased leptin protein content within adipose tissue of rats despite decreasing serum levels, emphasizing that circulating and tissue leptin expression may not be coordinately regulated [104]. In humans, serum leptin was also decreased following acute alcohol, although nutrient status and time of day influenced the results [105]. Therefore, leptin levels both in serum and adipose tissue are variable after chronic alcohol intake and at this time do not appear central to the regulation of adipose tissue metabolism by alcohol.

### 5.3. Resistin

Resistin can suppress adiponectin secretion as well as stimulate lipolysis to promote the inappropriate release of fatty acids and glycerol into the circulation [106]. Chronic alcohol feeding in rats increased serum resistin [16,17,24]. Similarly, chronic alcohol consumption by men increased resistin and 7 days of abstinence did not normalize this response [81]. In contrast, no change in resistin was detected in women alcoholics [81]. Serum concentrations were also not altered by three weeks of moderate alcohol intake (three cans of beer per night) [80]. Adipose tissue mRNA expression of resistin in rats (4 weeks of alcohol) did not differ from control values, while the protein content was increased [16,17]. However, a longer period of alcohol feeding (22 weeks) provided evidence that high dose alcohol (5 g/kg/day) increased resistin in visceral adipose tissue, while lower doses (0.5 g/kg/day and 2.5 g/kg/day) did not [24]. Although data are limited, a sufficiently high dose of chronic alcohol appears to be required to increase serum and adipose tissue resistin; however, the direct effects of this increase remain to be defined in the context of alcoholic disease both within the adipose tissue and in peripheral organs.

### 5.4. Chemerin and Visfatin

A lesser known adipokine, chemerin, has important autocrine and paracrine roles in the regulation of adipogenesis and adipocyte differentiation [107]. In humans and rats, higher levels of chronic alcohol intake increased chemerin in both serum and visceral adipose tissue [31]. Levels of chemerin in these men consuming alcohol were positively correlated with BMI, body fat levels, and triglycerides [31].

Visfatin is implicated in glucose metabolism within adipose tissue and other organ systems. A dose-dependent relationship was reported between chronic alcohol exposure and visfatin expression in visceral adipose tissue and serum of rats [24], where a daily dose of 5 g/kg/day was needed to increase plasma visfatin concentrations. Accordingly, acute alcohol intoxication at a relatively lower dose (2.5 g/kg) did not alter serum levels of visfatin in rats [89], but continuing this dose of alcohol for 3 days did decrease peptide levels in plasma [66].

Mounting evidence supports an effect of chronic alcohol on the major adipokines, leptin, and adiponectin. However, these effects are not entirely consistent when comparisons are made between rodents and humans. As these adipokines have pleiotropic actions throughout the body, establishing exactly how different doses of alcohol alter their actions will be important in future work. Further, most of the animal work reported involved only males and sex differences may exist, reiterating the need to include sex as a biological variable. A summary of the alcohol-induced changes in adipokine changes in various models is presented in Table 1.

## 6. Inflammatory Cytokines

Not surprisingly, alcohol increases the expression and release of several pro-inflammatory mediators (e.g., TNFα, IL-6, MCP-1) within and from adipose tissue that may contribute to the observed metabolic disturbances. For example, TNFα, can cause peripheral and hepatic insulin resistance [108]; accordingly, upregulation of TNFα and IL-6 by chronic alcohol was associated with impaired insulin-mediated glucose uptake by WAT [41]. TNFα also impairs adipose tissue metabolism by increasing HSL and lipolysis, and decreasing LPL and lipogenesis. Lastly, TNFα mRNA content is inversely associated with adiponectin mRNA in visceral adipose tissue following alcohol consumption [51].

Accordingly, adipose tissue expression of TNFα is consistently increased [12,15,18,22,30,41,51,64,71,86,103] in alcohol models lasting longer than 18 days at which time no change is noted [50,71]. This upregulation was observed in vitro (OP9 mouse stromal cells, 100 mM alcohol) [22] as well as in epididymal, perirenal, visceral, and subcutaneous adipose tissue depots [12,15,18,22,30,41,51,64,66,71,86,103]. Consistent with this apparent time-dependency, repeated binge-like alcohol (2.5 g/kg/day) for 3 days in rats did not change TNFα concentration in subcutaneous or mesentery adipose tissue [66]. In patients with acute alcoholic hepatitis, TNFα levels in subcutaneous adipose tissue were a stronger indicator of liver injury and systemic inflammation than TNFα produced by the liver [30]. Conversely, TNFα mRNA expression did not differ between patients with mild versus severe liver disease showing little predictive potential of this marker on liver status [87]. Lastly, one week of alcohol withdrawal in chronic alcoholic patients was not sufficient to reduce TNFα indicating the sustained inflammatory state is not immediately reversible [22].

The upregulation of TNFα expression is related to the metabolism of alcohol via CYP2E1, as the chronic alcohol-induced increase in TNFα was prevented in mice deficient for this enzyme [71]. Activation of PPARγ via treatment with rosiglitazone also prevented the alcohol-induced increase in TNFα, possibly due to the effects of the drug on fat metabolism and/or its anti-inflammatory actions that include suppression of nuclear factor-κB [12,51]. In this regard, the heightened inflammatory state in adipose tissue detected after 8 weeks of alcohol feeding was associated with increased expression of this transcription factor [18].

Similarly, IL-6 expression is also increased by chronic alcohol intake in rodents in WAT [12,18,22,41,50,64,71] and mesenteric adipose tissue [66]; however, it was undetectable after chronic binge alcohol (2.5 g/kg/day, 3 days) in subcutaneous adipose tissue of rats [66]. Further, IL-6 expression in alcoholics was indicative of more severe liver disease, was correlated positively with other inflammatory mediators [IL-18, osteopontin (OPN)] and fibrotic markers [α-smooth muscle actin (α-SMA) and semaphorin 7A (SEMA7A)], and remained elevated after one week of alcohol withdrawal [87].

Another major pro-inflammatory mediator, MCP-1 is also increased in chronic alcohol-fed mice [71] and rats [12,50,64,71], while to our knowledge, it has not been measured in human adipose tissue. Comparable to the findings for TNFα, rosiglitazone prevented the alcohol-mediated increase in MCP1 [12], as did the knockout of CYP2E1 [71]. As its name implies, MCP-1 recruits macrophages to sites of inflammation typically produced by tissue necrosis. Chronic alcohol leads to apoptosis within adipose tissue as evidenced by an increased number of deoxynucleotidyl transferase dUTP nick end labeling (TUNEL)-stained nuclei [71], although another investigation found no change in makers of the caspase pathway including caspase-3, total and cleaved poly ADP ribose polymerase (PARP), cleaved caspase-9, and B-cell lymphoma 2 (Bcl-2) [15]. This response appears related to inflammatory activation as inhibition of apoptosis in BH3 interacting-domain death agonist (Bid) knockout mice partially prevented the alcohol-induced increase in MCP-1 and pro-inflammatory macrophage marker CD11c [71].

Macrophages comprise almost half of the total immune cell population in adipose tissue making them an important component of adipose tissue signaling. Macrophages are typically visualized in adipose tissue as crown-like structures surrounding necrotic adipocytes for engulfment. In response to chronic alcohol intake of at least 25 days crown-like structures and CD11c [71] were increased as was the macrophage-specific cell surface glycoprotein ED2 [64]. However, F4/80 mRNA, a general macrophage marker, was not as sensitive to the potential inflammatory changes and was unchanged in this rodent model at both 18 and 25 days of alcohol intake [64].

Much attention has been paid to identify the macrophage phenotype (M1, M2) within adipose tissue as a means to determine the inflammatory profile. The pro-inflammatory M1 macrophages express inducible nitric oxide synthase (iNOS), TNFα, IL-1β, and CD11c, while M2 macrophages are characterized by IL-10 and arginase [109]. As discussed, chronic alcohol increased TNFα and CD11c expression in adipose tissue, while IL-1β is also increased after chronic alcohol in mice [15]. Further, interferon gamma (IFNγ) which can induce M1 polarization, was increased in WAT after chronic alcohol intake [103]. Therefore, despite no reports of iNOS expression in adipose tissue, it can be inferred that alcohol likely enhances the M1 polarization of macrophages similar to that observed during obesity. In relation to M2 activation, IL-10 mRNA in subcutaneous adipose tissue of alcoholic patients was increased, suggesting possible compensatory anti-inflammatory activity, but the limited data precludes definitive conclusions [30].

Recently, Fulham and Mandrekar, 2015 used the National Institute on Alcohol Abuse and Alcoholism (NIAAA) model of chronic-binge alcohol intoxication to investigate potential sex differences in adipose tissue inflammation and macrophage expression [110]. Their work showed that 10 days of alcohol and a single (5 g/kg) binge increased macrophage activation in female mice as evidenced by increased mRNA expression of F4/80, CD68, CD11b, and CD11c. However, no significant change was observed in TNFα, IL-6, MCP1 mRNA, or the appearance of crown-like structures within the adipose tissue, possibly due to the short duration of the alcohol feeding [110]. Therefore, the increase in macrophage markers observed at this early time point may represent an initiating factor in the upregulation of other inflammatory or lipolytic/lipogenic mediators.

A mechanism through which alcohol produces a pro-inflammatory state within adipose tissue has been proposed by Sebastian et al., 2011 [71]. The induction of CYP2E1 by alcohol is implicated in adipocyte apoptosis via TNFα, and apoptosis leads to the activation of the classical complement pathway via binding of C1q to apoptotic adipocytes. Products of complement activation bind to the C3a receptor and C5a receptor to cause enhanced cytokine and chemokine release (i.e., development of pro-inflammatory state). Outside of this mechanistic work it remains to be determined if other pathways are altered by chronic alcohol in the regulation of inflammatory mediators.

As cited above, prolonged alcohol intake is required to stimulate production of inflammation and accordingly, acute alcohol intoxication has not been found to alter the pro-inflammatory mediators in adipose tissue. For example, an acute dose of alcohol (1.12 g/kg) or episodic (3 days 1.12 g/kg; 2–4 days no alcohol; 3 days 1.12 g/kg) treatment did not altered the expression of TNFα, IL-6, MCP-1, neutrophil chemokine keratinocyte chemoattractant (KC), elastase, IL-1β, or IL-10 [90]. However, these effects may be depot- and dose-specific as a chronic binge-like alcohol protocol (2.5 g/kg/day, 3 days) in male rats increased IL-1α, IL-1β, IL-6, granulocyte-macrophage colony-stimulating factor (GM-CSF), CD11c, CD4, and mast cells in mesentery adipose tissue, but did not in subcutaneous adipose tissue [66].

The inflammatory environment generated by chronic alcohol consumption provides an interesting paradigm of study, as it mimics several aspects of obesity including an increase in pro-inflammatory factors and macrophage recruitment; however, it does so without significant adipose tissue expansion. Additionally, similar to obesity is the upregulation of factors like TNFα and IL-6 that are implicated in the development of insulin resistance and glucose intolerance. Lastly, the induction of the pro-inflammatory environment likely contributes to the alcohol-induced increase in lipolysis and ectopic lipid storage, although the mechanism through which alcohol exerts its actions on the adipose tissue remain to be fully elucidated, but perhaps future hypotheses can be generated from the much larger obesity-related data set.

## 7. Brown Adipose Tissue

Brown adipose tissue is distinctly different from WAT in form and function. BAT contains multiple small lipid droplets (multilocular) and a centralized nucleus, whereas WAT contains a single large lipid droplet (i.e., unilocular) and a peripherally located nucleus. Most importantly, BAT has a larger number of mitochondria that are responsible for its high metabolic rate and heat generating properties. Alcohol easily permeates BAT as measurable levels have been reported in alcohol-fed mice [45]. Compared with the liver, BAT has a low level of ADH activity which is unchanged after 10 days of chronic alcohol intake [72,111].

The effects of alcohol on BAT mass of the interscapular depot are inconsistent as no change, an increase and decrease have all been reported depending on the experimental conditions [72,112,113,114]. No change in BAT mass has been observed after 10 days (male mice) [72], 14 days (male rats) [112], or 5 weeks (mice) [46] of alcohol, while one report observed a decrease after 25 days (male mice) [72]. In mice categorized as being obese (body weight (BW) > 40 g), 5 weeks of alcohol in the drinking water prevented the increase in BAT observed in the control mice, suggesting that alcohol may suppress expansion of this fat depot during periods of over-nutrition [46]. Conversely, prolonged alcohol consumption increased BAT weight in rats (10% *v*/*v* in drinking water) [113] as well as in pups born to alcohol consuming dams [114]. Protein [72,113,114] and DNA [72] content in BAT after alcohol paralleled changes in BAT weight, regardless of the directional change. Therefore, it appears that alcohol intake must be of sufficient length to induce an adaptive response.

Enhanced abundance of mitochondria within BAT modulates the increase in thermogenesis as production of ATP by oxidative metabolism is uncoupled from ATP generation by uncoupling protein 1 (UCP-1). Oxidative enzyme activity of cytochrome oxidase and succinate dehydrogenase was increased in isolated mitochondria from BAT of rats consuming alcohol for 6.5 months (10% *v*/*v* in water), and in pups from an alcohol-dosed dam [113]. In contrast, shorter durations of alcohol suppressed the increase in succinate dehydrogenase caused by single housing the animals during a 10- and 25-day experimental alcohol feeding regime [72]. As with BAT size, adaptive changes in mitochondrial activity results from longer duration alcohol intake.

Thermogenesis in BAT following alcohol has been superficially assessed in early work and supports an alcohol effect. For instance, denervation of BAT in alcohol-fed rats enhanced thermogenesis which could represent a mechanism for the lack of body weight gain after prolonged alcohol consumption [115]. This evidence, however, is inferential at best. Another experiment showed that survival time in cold conditions (−20 °C) was lengthened following chronic alcohol feeding but shortened by an acutely intoxicating dose of alcohol given immediately before exposure, suggesting that chronic alcohol increased BAT and thereby thermogenesis [116]. Early biochemical based detection of thermogenesis is also somewhat inconclusive; in isolated mitochondria from BAT, alcohol (14 days, 7% *v*/*v* alcohol in water, rats) increased specific guanosine diphosphate (GDP) binding indicating uncoupled respiration and thermogenesis within the mitochondria [112]. However, these results have not been replicated as consumption of a slightly higher level of alcohol (10% *v*/*v* in water) for 10 and 25 days decreased GDP binding in isolated mitochondria [72]. Therefore, early work neither convincingly supports nor refutes the potential effects of alcohol on thermogenesis in BAT.

In extension of these findings, UCP-1 the primary protein involved in thermogenesis within BAT has also been measured in response to alcohol. UCP-1 shuttles H^+^ from the electron transport chain across the mitochondrial membrane without allowing ATP synthesis to occur and instead heat from the metabolized energy is produced. Initial studies showed the relative abundance of UCP-1 protein in isolated mitochondria from BAT was decreased after chronic alcohol consumption for 10 or 25 days (10% *v*/*v* in drinking water, male mice) [72], suggesting a decrease in the thermogenic capacity of BAT. In contrast, UCP-1 mRNA content of interscapular BAT following acute alcohol intoxication was variable and time- and dose-dependent. For example, mRNA of UCP-1 was not changed by a 2 g/kg dose [117] at early (30 min, 1 h) and later time points (8 h, 16 h), but was increased at 2 and 4 h post intoxication [118]. Similarly, a much lower dose of alcohol (0.5 g/kg) did not change UCP-1 mRNA at 30 min, 2 h, 8 h, and 16 h but decreased it at 1 h and increased it at 4 h [118]. Dose-dependent effects of alcohol on UCP-1 were confirmed as a 2 g/kg dose had higher UCP-1 expression than a 0.5 g/kg dose [118] and in a separate study no effect of 2 g/kg on UCP-1 was observed, while a 3 g/kg dose led to decreased expression [117]. While these findings exemplify what appears to be the dose-dependent and transient nature of UCP-1 expression after alcohol, they do little to resolve whether alcohol leads to a sustained change in thermogenesis per se as the only chronic investigation was of relatively short duration (4 weeks) and has not been replicated.

Thermogenesis is activated by the sympathetic nervous system and in particular norepinephrine, which increases metabolic rate. Acute alcohol intoxication (2 g/kg and 3 g/kg) did not change norepinephrine when rats were kept at room temperature [117]. However, when exposed to 4 °C for 2 h following acute intoxication, alcohol suppressed the cold-induced increased in norepinephrine in BAT with the higher 3 g/kg dose having a greater effect [117].

As in WAT, alcohol modulates enzymes of lipolysis and lipogenesis in BAT. In general, acute and chronic alcohol decreases the activity of the lipolytic enzyme HSL in BAT and in primary BAT adipocytes [45,46,47]. Accordingly, cAMP accumulation was also consistently decreased by acute and chronic alcohol, indicating suppression of lipolysis via the β-AR system [45,46,47]. These findings indicate an alcohol-induced decrease in BAT lipolysis in favor of maintenance of BAT tissue mass, although there is an opposing report of a decrease in the lipogenic enzyme LPL in BAT after chronic alcohol [47]. In contrast, acute alcohol in vitro and in vivo does not alter LPL activity, implying that chronic consumption is required [47]. Interestingly, HSL activity and cAMP accumulation were increased upon withdrawal of alcohol after chronic feeding, compared with control values, for 3–12 h before normalizing to baseline levels, suggesting an immediate physiological compensation within BAT [45].

BAT thermogenesis relies on lipolysis and free fatty acid availability either via UCP-1 activation or directly as a fuel source. It is also a primary tissue for clearing lipids from plasma in rodents. Therefore, similar to the liver, it is likely that the lipolytic effects of alcohol on WAT may contribute to the changes observed in BAT. Increased lipolysis of WAT by chronic alcohol would increase the availability and uptake of FFAs by BAT, thereby enhancing BAT thermogenesis. In obese populations, this effect could potentially be efficacious; however, in a disease commonly accompanied by under-nutrition, the alcohol-induced increase in thermogenesis may promote weight loss, both WAT mass and lean mass loss, and subsequently worsen health status. However, it remains to be determined whether fatty acids from WAT are taken up by BAT during chronic alcohol intake, suggesting a topic for future study.

Overall, the effects of alcohol on BAT remain somewhat enigmatic, and additional research is required now that our knowledge of BAT function and regulation has significantly advanced from the time when the original studies were conducted. Aside from the obvious need to extend work to include females and other BAT depots, as interscapular BAT accounts for only ~20–25% of total BAT, additional studies should focus on establishing whether alcohol does in fact have a thermogenic effect and the consequences thereof. Perhaps pharmacological regulation of thermogenesis (if altered in alcoholics) could assist in correcting some of the metabolic defects induced in both BAT and WAT and resulting consequences.

## 8. Conclusions

**Figure 3:**
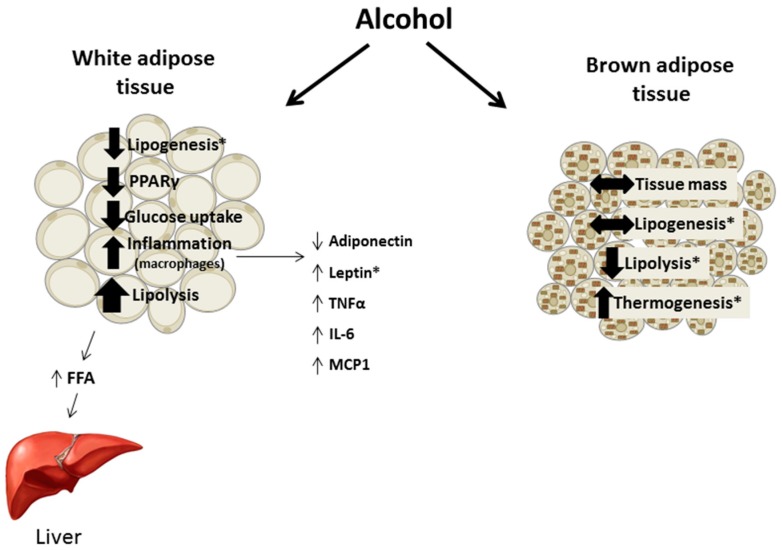
Summary of the effects of chronic alcohol on white and brown adipose tissue function. In white adipose tissue, alcohol leads to overall loss of tissue mass due to enhanced lipolysis and either unchanged or decreased lipogenesis. FFAs released following lipolysis are commonly deposited in the liver contributing to alcoholic steatosis. Glucose uptake is also perturbed by alcohol. A heightened inflammatory state develops from chronic alcohol consumption leading to increased macrophage infiltration and expression of the indicated adipokines/cytokines. Within brown adipose tissue conclusive data are limited but suggest a lack of change in tissue mass and lipogenesis as well as a decrease in lipolysis and potential increase in thermogenesis. * Indicates where findings have been variable and inconclusive but suggest the indicated effect. Abbreviations: peroxisome proliferator activated receptor γ (PPARγ), tumor necrosis factor α (TNFα), interleukin-6 (IL-6), and monocyte chemoattractant protein-1 (MCP1).

Chronic alcohol intake alters adipose tissue metabolism including inappropriate activation of lipolysis, impaired insulin-mediated glucose uptake, and perturbations in adipokine secretion and expression leading to the promotion of an inflammatory environment (Figure 3). These effects are not exclusive to the adipose tissue as the lipids released by lipolysis contribute to hepatic steatosis and adipokines can affect tissues throughout the body. Throughout the review methodological differences potentially contributing to divergent findings were included, although they do not always fully explain inconsistencies in the results. For example, feeding status and hence the nutritional state of the animal is often not clearly presented. Further, few animal studies included females in their experimental design and in the studies that did, their results often differed from data in males, suggesting that sex hormones may influence the response of adipose tissue to alcohol. As women are more prone to several alcohol-related diseases and may have a different distribution and proportion of adipose tissue, examining sexual dimorphic responses in a systematic manner will likely provide valuable data advancing the field. Overall, the effects of alcohol on adipose tissue merit further study especially in relation to other organ systems negatively impacted in alcohol use disorder.

## Figures and Tables

**Table 1:** Modulation of adipokines by alcohol. The effects of either acute or chronic alcohol on circulating adipokine levels or expression within adipose tissue are listed below along with the general physiological action of the given adipokine. Note that the given action may or may not have been confirmed to occur following alcohol. Arrows indicate the direction of change reported under the given conditions.

Adipokine	Action	Plasma Acute Alcohol Chronic Alcohol				Adipose Tissue Acute Alcohol Chronic alcohol			
		Rodent	Human	Rodent	Human	Rodent	Human	Rodent	Human
Adiponectin	Adipogenic; insulin sensitizer	↓ [89]		↓ [9,18,49,50,51,62,74,75,76,77]	↑ [36,37,79,80,81,82,83,84]	↔ [90]		↓ [15,18,24,25,49,51,74,86].	↑ [37]
		↔ [66]		↔ [10,12,25,78]	↓ [34,35]			↑ [16]	
Leptin	Lipolysis, fatty acid oxidation, lipogenesis, insulin sensitivity; responsive to changes in fat mass	↓ [104]	↓ [105]	↓ [10,93,94]	↓ [27,101]	↑ [104]		↑ [16,22,24,103]	↔ [30]
				↔ [24]	↔ [80,87,95,96,97,98,99]				
				↑ [24,91,92]	↑ [100,102]				
Resistin	Stimulate lipolysis and fatty acid release, suppress adiponectin			↑ [16,17,24]	↑ [81]			↔ [16,17,24]	
					↔ [80,81]			↑ [16]	
Chemerin	Adipogenesis and adipocyte differentiation			↑ [31]	↑ [31]			↑ [31]	↑ [31]
Visfatin	Glucose metabolism	↔ [89]		↑ [24]					
				↔ [24]					
